# AlzPathway: a comprehensive map of signaling pathways of Alzheimer’s disease

**DOI:** 10.1186/1752-0509-6-52

**Published:** 2012-05-30

**Authors:** Satoshi Mizuno, Risa Iijima, Soichi Ogishima, Masataka Kikuchi, Yukiko Matsuoka, Samik Ghosh, Tadashi Miyamoto, Akinori Miyashita, Ryozo Kuwano, Hiroshi Tanaka

**Affiliations:** 1 Department of Bioinformatics, Tokyo Medical and Dental University, Yushima 1-5-45, Bunkyo-ku, Tokyo, 113-8510, Japan; 2 Systems Biology Institute, Shirokanedai 5-6-9, Minato-ku, Tokyo, 108-0071, Japan; 3 JST ERATO Kawaoka Infection-induced Host Response Network Project, The Institute of Medical Science, University of Tokyo, Bld 2, 4F, 4-6-1 Shirokanedai, Minato, Tokyo, 108-8639, Japan; 4 Department of Molecular Genetics, Center for Bioresources, Brain Research Institute, Niigata University, Niigata, 951-8585, Japan

## Abstract

**Background:**

Alzheimer’s disease (AD) is the most common cause of dementia among the elderly. To clarify pathogenesis of AD, thousands of reports have been accumulating. However, knowledge of signaling pathways in the field of AD has not been compiled as a database before.

**Description:**

Here, we have constructed a publicly available pathway map called “AlzPathway” that comprehensively catalogs signaling pathways in the field of AD. We have collected and manually curated over 100 review articles related to AD, and have built an AD pathway map using CellDesigner. AlzPathway is currently composed of 1347 molecules and 1070 reactions in neuron, brain blood barrier, presynaptic, postsynaptic, astrocyte, and microglial cells and their cellular localizations. AlzPathway is available as both the SBML (Systems Biology Markup Language) map for CellDesigner and the high resolution image map. AlzPathway is also available as a web service (online map) based on Payao system, a community-based, collaborative web service platform for pathway model curation, enabling continuous updates by AD researchers.

**Conclusions:**

AlzPathway is the first comprehensive map of intra, inter and extra cellular AD signaling pathways which can enable mechanistic deciphering of AD pathogenesis. The AlzPathway map is accessible at http://alzpathway.org/.

## Background

Alzheimer’s disease (AD) is the most common cause of dementia among the elderly. With the advent of the aging society, 24.3 million people are estimated to suffer dementia worldwide, increasing to 42.3 million people by 2020 and 81.1 million by 2040 [1,2]. The total estimated worldwide costs of dementia are US$604 billion in 2010, and are estimated to increase by 85% to 2030 [3]. The cost for caring for the increasing number of people with dementia will rise dramatically and thus will be disastrous burden to our societies within upcoming 10–20 years. To address this social issue, clarification of the pathogenic mechanism of AD and development of AD drugs are urgently expected.

Genetic association with putative AD susceptibility genes has been studied and collected as a publicly available database called AlzGene (http://www.alzgene.org/) [4]. Pathological signaling has been also studied and two core pathological hallmarks of AD, amyloid plaques and neurofibrillary tangles (NFT) accumulation, have been elucidated intensively. However, wealth of this information has become increasingly difficult to follow, much less interpret, and has not been integrated before. Integration of pathway knowledge bridging amyloid plaques and neurofibrillary tangles has been still missing.

Efforts to construct an AD pathway map have been made before, however, these maps are overviews or partial maps of an AD pathway, and are not comprehensive maps. Manual elaboration is still required to construct a comprehensive and accurate map of a particular signaling pathway [5]. Efforts to manually construct pathway maps of particular signaling, e.g., Toll-like receptor, EGFR, RB/E2F, mTOR signalings, and dendritic cell signaling in response to pathogenes, have been made before [6-10].

In this study, we collected and manually curated over 100 review articles involving in AD, and built an AD pathway map using CellDesigner [11], a modeling editor for biochemical pathways. AlzPathway is the first comprehensive map of intra, inter and extra cellular signaling pathways of particular disease. AlzPathway now contains 34 canonical pathways such as APP, mitochondrial and apoptosis pathways, which are composed of 1347 species (proteins, complexes, simple moecules, genes, RNAs, ions, degraded products, and phenotypes), 1070 reactions, 129 phenotypes in neuron, brain blood barrier, presynaptic, postsynaptic, astrocyte, and microglial cells and their cellular localizations. Our AlzPathway provides a powerful AD pathway map for deciphering pathogenesis of AD, and it serves as a pathway reference database just as AlzGene for risk-gene reference database. AlzPathway allows us to not only evaluate candidate risk genes listed by GWAS studies, but also analyze omics data including DNA microarray data and RNA-seq data to reveal pathogenesis of AD. Our pathway map will be an indispensable basic resource for both clarification of the pathogenic mechanism of AD and development of AD drug to address social issues caused by AD.

## Construction and content

### Construction of AlzPathway

We collected 123 review articles related to AD accessible from PubMed. We then manually curated these review articles, and have built an AD pathway map by using CellDesigner. In PubMed, the number of research articles involved in Alzheimer’s disease is over 80,000. In fact, we cannot collect and manually curate over 80,000 research articles. To construct AlzPathway, we need to choose articles among over 80,000 research articles. Review articles are attempts to chose important articles and summarize current state of understanding on signaling pathways involved in Alzheimer’s disease. Therefore, we chose to use review articles for the curation. Notations are based on the PD (Process Description) of SBGN (Systems Biology Graphical Notation) [12] and the map is available in standardized SBML (Systems Biology Markup Language) [13] format for file exchange between different tools. Molecules are distinguished by the following types: proteins, complexes, simple molecules, genes, RNAs, ions, degraded products, and phenotypes. Gene symbols are pursuant to the HGNC symbols. Reactions are also distinguished by the following categories: state transition, transcription, translation, heterodimer association, dissociation, transport, unknown transition, and omitted transition. All the reactions have evidences to the references in PubMed ID using the MIRIAM scheme [14]. All the references used for constructing the AlzPathway are listed in the ‘References for AlzPathway’. Cellular types are distinguished by the followings: neuron, astrocyte, and microglial cells. Cellular compartments are also distinguished by the followings: brain blood barrier, presynaptic, postsynaptic, and their inner cellular localizations. AlzPathway is available as both the SBML map for CellDesigner ( Additional file 1; see the section of Additional files) and the high resolution image map ( Additional file 2; see the section of Additional files).

### Imprementation of web service of AlzPathway

AlzPathway is also available as the web service (online map) implemented by using Payao [15], a community-based, collaborative web service platform for gene-regulatory and biochemical pathway model curation, enabling continuous updates by AD researchers. Payao web service (online map) is accessible from http://alzpathway.org/. Instruction on how to access the AlzPathway web service (online map) is described in the Additional documentation file ( Additional file 3; see the section of Additional files). Using the Payao system would enable AD researchers not only to browse reactions and their references in PubMed ID but also to comment, correct and update AlzPathway in a community-wide collaboration.

### Overview of alzheimer’s disease pathway

**Figure 1:**
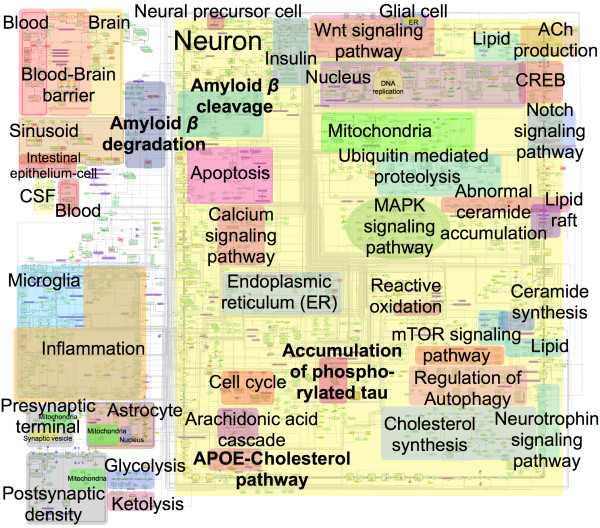
**Overview of AlzPathway overlaid with canonical pathway annotations for explanation of map.** AlzPathway consists of 1347 molecules, 1070 reactions, and 129 phenotypes. Original and high resolution version of AlzPathway is available at http://alzpathway.org/ as both the SBML (Systems Biology Markup Language) map for CellDesigner and the high resolution image map without overlay of canonical pathway annotation. AlzPathway is also available as the web service (online map) implemented by using Payao system.

Here, we present a map of Alzheimer’s disease signaling networks (Figure 1). We manually constructed a map of Alzheimer’s disease signaling networks by assembling molecular interactions based on published papers using the modeling software, CellDesigner ver. 4.2 (http://celldesigner.org/).

**Figure 2:**
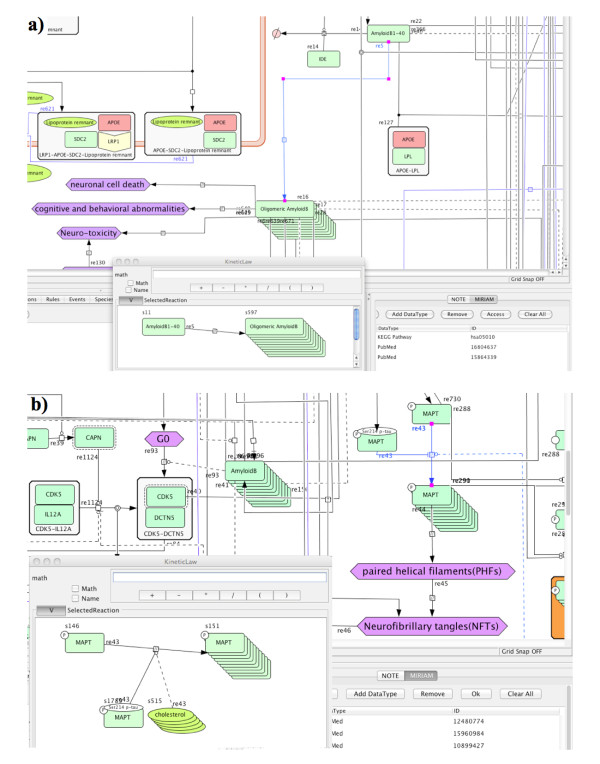
**Detailed view of AlzPathway. (a) oligomeric amyloid β formation, (b) MAPT phosphorylation.** Notations are based on SBGN (Systems Biology Graphical Notation). References to the reactions are represented in PubMed ID (PMID) using the MIRIAM scheme.

The AlzPathway map consists of 1347 species, 1070 reactions, and 129 phenotypes. The molecues shown on the AlzPathway can be categorized as follows: 650 proteins, 232 complexes, 223 simple molecules, 32 genes, 36 RNAs, 24 ions, and 21 degraded products. The breakdown of reactions is as follows: 401 state transitions, 22 transcriptions, 30 translations, 172 heterodimer associations, 49 dissociations, 87 transports, 20 unknown transitions, and 228 omitted transitions. All the 123 references used for constructing the map are listed in the ‘References for AlzPathway’ [16-138]. The CellDesigner software allows the user to access references that are used as evidences for individual reaction using PubMed ID (Figure 2).

The map consists of the AD hallmark pathways and canonical pathways. The AD hallmark pathways are amyloid β cleavage, amyloid β degradation, APOE-cholesterol pathway and NFT accumulation, which are major pathological pathways of AD. On the other hand, the canonical pathways are acetylcholine production, cholesterol synthesis, Wnt signaling pathway, Notch signaling pathway, Ubiquitin mediated proteolysis, apoptosis, calcium signaling pathway, ER stress, MAPK signaling pathway, abnormal ceramide accumulation, ceramide synthesis, reactive oxidation process, regulation of Autophagy, neurotrophin signaling pathway, cell cycle, arachidonic acid cascade, mTOR signaling pathway, lipid pathway, lipid raft, inflammation pathway, insulin pathway, and CREB pathway.

AlzPathway is the first comprehensive map of intra, inter and extra cellular signaling pathways of particular disease manually constructed. Manual reconstructions of comprehensive map have been reported before: epidermal growth factor receptor signaling, toll-like receptor signaling network, RB/E2F signaling pathway and mTOR signaling pathways [6-9], which are molecular signaling pathways and are not comprehensive intra, inter and extra cellular signaling pathways of particular disease. That is, AlzPathway comprehensively catalogs not only intra but also inter and extracellular signaling pathways among neuron, glial cell, microglia, presynaptic cell, postsynaptic cell, astrocyte, and blood–brain barrier. The brain and spinal cord are made up of various regions and cells, including neurons and glial cells. To reveal pathogenic mechanism of AD, complicated signaling pathways among neuron, glial cell, microglia, presynaptic cell, postsynaptic cell, astrocyte, and blood–brain barrier should be clarified.

### Binary-relation notation and key molecules

**Figure 3:**
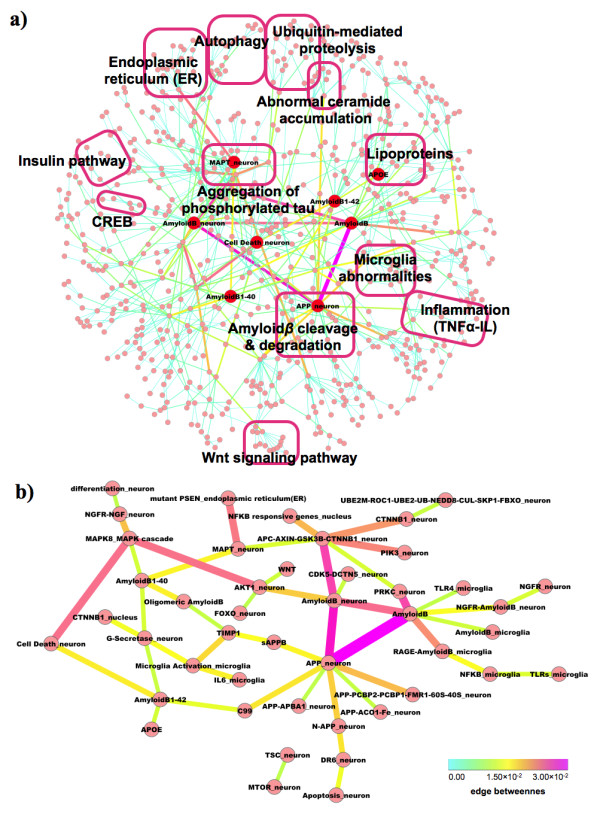
**Binary-relation notation and key molecules of AlzPathway.** (**a**) overview of AlzPathway in binary-relation notation, (**b**) top 50 high centrality relations as highlighted primary pathway of AlzPathway.

As for intuitively understandable notation of AlzPathway, we also constructed AlzPathway not only in SBGN (Systems Biology Graphical Notation) PD (Process Description) notation but also in binary-relation notation (Figure 3(a)). In SBGN PD notation, a reaction consists of reactants, modifiers, and products. We converted this notation to binary-relation notation by decomposing reactions into those between (1) reactants and products, and (2) modifiers and products. Molecules are limited to proteins, complexes, genes, RNAs and phenotype for simplification. SBGN PD notation is precise notation for describing pathways, on the other hand the binary-relation based conventional notation used by molecular biologists in the current literature, is intuitively understandable. To clarifying basic structure of AlzPathway, we constructed AlzPathway in binary-relation notation, and found that amyloid β accumulation and hyperphosphorylated tau accumulation are central pathogenesis signaling pathways in AlzPathway. Binary-relation notation is provided as a SIF (simple interaction format) file ( Additional file 4) which can be opened by using Cytoscape [139].

According to edge betweenness centrality of each reaction, high centrality relations were highlighted as primary relations. Top 50 high centrality relations are shown in Figure 3(b). Highlighted binary relations were so-called AD hallmark pathways: amyloid plagues (amyloid β accumulation) and NFT accumulation (hyperphosphorylated tau accumulation). The γ-secretase produces amyloid β 1–40, which leads to oligomeric amyloid β (amyloid β accumulation). Amyloid β 1–42 is related to cell death, which is amyloid plagues (amyloid β accumulation) and NFT accumulation crucial for AD progression. On the other hand, APC-AXIN-GSK3B-CTNNB1 complex phosphorylates MAPT and mutant PSEN promotes phosphorylation of MAPT, which lead to MAPT hyperphosphorylation and NFT accumulation.

## Utility and discussion

### Access to AlzPathway map

The AlzPathway map is accessible at http://alzpathway.org/. As described above, AlzPathway is provided as (1) the SBML map for CellDesigner ( Additional file 1), (2) the web service (online map) implemented by using Payao accessible from http://alzpathway.org/ (see the Additional file 3 for instruction on how to access the AlzPathway online map by Payao), (3) the high resolution image map ( Additional file 2), (4) the binary-relation notation ( Additional file 4), (5) the pure SBML map ( Additional file 5) for compatibility with other SMBL supporting tools, and (6) the BioPAX file for exchanges of pathway data ( Additional file 6). For Notations are based on the PD (Process Description) of SBGN (Systems Biology Graphical Notation). The CellDesigner software and Payao system allow the user to access references that are used as evidences for individual reaction in PubMed ID using the MIRIAM scheme. They also allow the user to access external resources including UniProt and PubChem for individual species using the MIRIAM scheme. AlzGene information is provided for individual species using Notes. Expanded version of AlzPathway map having external links to Gene Ontology is also provided ( Additional file 7). As for usage of CellDesigner software, see the documentations provided at CellDesigner web site. As for usage of Payao system, see the user’s guide provided at Payao web site.

### Community driven update of AlzPathway using payao

**Figure 4:**
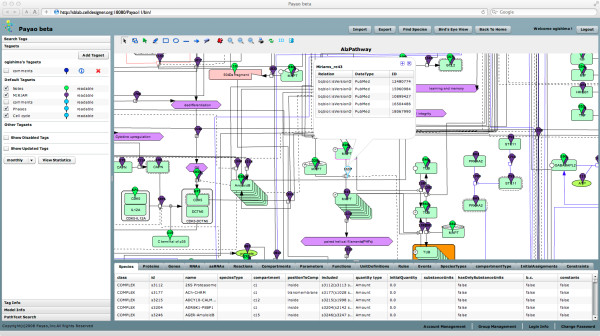
AlzPathway on Payao system. Payao system provide community-based, collaborative web service (online map) platform for pathway manual curation.

We constructed AlzPathway to be comprehensive but not necessarily to be complete. While continuous curation of molecular pathways remain a major challenge, the availability of Alzpathway through Payao (http://alzpathway.org/) would enable AD researchers to review, comment and provide feedbacks to the pathway through the web-based interface (Figure 4). Correction and update of AlzPathway can be made through comments and feedback from AD researchers in specific molecules and interactions. By using Payao system, we envision to reach out to the AD community and continuously correct and update AlzPathway. In addition to community-driven correction and update using Payao system, we will keep updating our pathway map by ourselves.

### AlzPathway as basic resource for AD study

AlzPathway is comprehensive AD pathway map, and is expected to be a guidance map in the study of AD. Just as AlzGene for risk-gene reference database, AlzPathway serves as a pathway “reference” integrating wealth of pathway information published before. AlzPathway also serves for analysis on omics data including DNA microarray data and RNA-seq data. Availability of AD pathway map in standardized formats (SBML and SBGN) renders the network applicable to systems-biology analyses based on various SBML compliant tools. Therefore, our AD pathway map will be an indispensable basic resource for both clarification of the pathogenic mechanism of AD and development of AD drug.

## Conclusions

We constructed a publicly available database called “AlzPathway” that comprehensively catalogs signaling pathways in the field of AD. We have collected and manually curated over 100 review articles involving in AD, and have built an AD pathway map using CellDesigner. AlzPathway is the first comprehensive map of intra, inter and extra cellular signaling pathways of particular disease for deciphering pathogenesis of AD. AlzPathway is currently composed of 1347 molecules, 1070 reactions, and 129 phenotypes in neuron, brain blood barrier, presynaptic, postsynaptic, astrocyte, and microglial cells and their cellular localizations. AlzPathway is available as both the SBML (Systems Biology Markup Language) map for CellDesigner and the high resolution image map. AlzPathway is also available as the web service (online map) implemented on Payao, a community-based, collaborative web service platform for pathway model curation. The molecular level mechanistic interactions captured in AlzPathway, together with a community-driven, web based curation platform, would provide a comprehensive resource to the AD community towards deeper insights into AD pathogenesis and identification of novel therapeutic targets.

## Availability and requirements

The AlzPathway map is accessible at http://alzpathway.org/. The Payao system does not work on a 64 bit mode Linux.

## Abbreviations

AD, Alzheimer’s disease; SBML, Systems biology markup language; NFT, Neurofibrillary tangles; PD, Process description; SBGN, Systems biology graphical notation.

## Competing interests

The authors declare that they have no competing interests.

## Authors’ contributions

SO conceived and designed the project with the help of RK and HT. SM, RI, TM, MK, and SO built the database. AM and RK reviewed the database. YM and SG implemented the web service by using Payao. SO wrote the paper with the help of YM, SG, MK, and TM. All authors read and approved the final manuscript.

## Supplementary Material

Additional file 1: **SBML map file of AlzPathway. **The SBML map file alzpathway_sbml_map.xml can be browsed using CellDesigner. Please download CellDesigner at http://www.celldesigner.org/, install it, and open the SBML map file alzpathway_sbml_map.xml to browse AlzPathway map by CellDesigner. As for usage of CellDesigner software, see the documentations provided at CellDesigner web site: http://www.celldesigner.org/documents.html. Click here for file

Additional file 2: **High resolution image map of AlzPathway. **The PNG file alzpathway_image_map.png contains a high resolution map of AlzPathway. This image map does not contain the reference information used for constructing the AlzPathway. SBML (CellDesigner) map and Online map (Payao) are recommended to browse the AlzPathway map. Click here for file

Additional file 3: **Instruction on usage of Payao. The PDF file alzpathway_payao_access_instruction.pdf contains insturction on usage of Payao web service (online map). **Payao system requires login. AlzPathway demo user account is prepared for demo use: demo username is “ap_demo” and the corresponding password is “4patients”. As for usage of Payao system, see the user’s guide provided at Payao web site: http://payao.oist.jp:8080/payaologue/doc/PAYAO_Users_GuideE11.pdf. Click here for file

Additional file 4: **Binary-relation notation file of AlzPathway. **The SIF (simple interaction format) file alzpathway_binary_relation.sif is the binary-relation notation of AlzPathway map which can be opened by using Cytoscape. Click here for file

Additional file 5: **Pure SBML map file of AlzPathway. **The pure SBML file alzpathway_pure_sbml.xml is for compatibility with other SMBL supporting tools. Click here for file

Additional file 6: **BioPAX file of AlzPathway.** The BioPAX file alzpathway_biopax.owl is for exchanges of pathway data. Click here for file

Additional file 7: **Expanded SBML map file of AlzPathway with Gene Ontology.** The SBML map file alzpathway_sbml_map_go.xml is the expanded version of AlzPathway map (alzpathway_sbml_map.xml) having external links to Gene Ontology. The SBML map file with the external links to Gene Ontology requires time to be opened by CellDesigner 4.2, and thus was provided separately as an expanded SBML map. Click here for file

## References

[B1] Ballard C Gauthier S Corbett A Brayne C Aarsland D Jones E Alzheimer’s disease Lancet 2011 377 9770 1019 1031 10.1016/S0140-6736(10)61349-9 21371747

[B2] Ferri CP Prince M Brayne C Brodaty H Fratiglioni L Ganguli M Hall K Hasegawa K Hendrie H Huang Y Global prevalence of dementia: a Delphi consensus study Lancet 2005 366 9503 2112 2117 16360788 10.1016/S0140-6736(05)67889-0 PMC2850264

[B3] Alzheimer’s Disease Interanational World Alzheimer’s Report 2010 http://www.alz.co.uk/research/files/WorldAlzheimerReport2010ExecutiveSummary.pdf

[B4] Bertram L McQueen MB Mullin K Blacker D Tanzi RE Systematic meta-analyses of Alzheimer disease genetic association studies: the AlzGene database Nat Genet 2007 39 1 17 23 10.1038/ng1934 17192785

[B5] Bauer-Mehren A Furlong LI Sanz F Pathway databases and tools for their exploitation: benefits, current limitations and challenges Mol Syst Biol 2009 5 290 19638971 10.1038/msb.2009.47 PMC2724977

[B6] Oda K Matsuoka Y Funahashi A Kitano H A comprehensive pathway map of epidermal growth factor receptor signaling Mol Syst Biol 2005 1 2005.0010 16729045 10.1038/msb4100014 PMC1681468

[B7] Oda K Kitano H A comprehensive map of the toll-like receptor signaling network Mol Syst Biol 2006 2 2006.0015 10.1038/msb4100057 PMC1681489 16738560

[B8] Calzone L Gelay A Zinovyev A Radvanyi F Barillot E A comprehensive modular map of molecular interactions in RB/E2F pathway Mol Syst Biol 2008 4 173 18319725 10.1038/msb.2008.7 PMC2290939

[B9] Caron E Ghosh S Matsuoka Y Ashton-Beaucage D Therrien M Lemieux S Perreault C Roux PP Kitano H A comprehensive map of the mTOR signaling network Mol Syst Biol 2010 6 453 21179025 10.1038/msb.2010.108 PMC3018167

[B10] Patil S Pincas H Seto J Nudelman G Nudelman I Sealfon SC Signaling network of dendritic cells in response to pathogens: a community-input supported knowledgebase BMC Syst Biol 2010 4 137 10.1186/1752-0509-4-137 20929569 PMC2958907

[B11] Funahashi A Matsuoka Y Jouraku A Morohashi M Kikuchi N Kitano H CellDesigner 3.5: a versatile modeling tool for biochemical networks Proc IEEE 2008 96 8 1254 1265

[B12] Le Novère N Hucka M Mi H Moodie S Schreiber F Sorokin A Demir E Wegner K Aladjem MI Wimalaratne SM The Systems Biology Graphical Notation Nat Biotechnol 2009 27 8 735 741 10.1038/nbt.1558 19668183

[B13] Hucka M Finney A Sauro HM Bolouri H Doyle JC Kitano H Arkin AP Bornstein BJ Bray D Cornish-Bowden A The systems biology markup language (SBML): a medium for representation and exchange of biochemical network models Bioinformatics 2003 19 4 524 531 10.1093/bioinformatics/btg015 12611808

[B14] Laibe C Le Novère N MIRIAM Resources: tools to generate and resolve robust cross-references in Systems Biology BMC Syst Biol 2007 1 58 10.1186/1752-0509-1-58 18078503 PMC2259379

[B15] Matsuoka Y Ghosh S Kikuchi N Kitano H Payao: A community platform for SBML pathway model curation Bioinformatics 2010 26 10 1381 1383 10.1093/bioinformatics/btq143 20371497 PMC2865864

[B16] Allain H Bentué-Ferrer D Tribut O Gauthier S Michel BF Drieu-La Rochelle C Alzheimer’s disease: the pharmacological pathway Fundam Clin Pharmacol 2003 17 4 419 428 10.1046/j.1472-8206.2003.00153.x 12914543

[B17] Arendt T Holzer M Stöbe A Gärtner U Lüth HJ Brückner MK Ueberham U Activated mitogenic signaling induces a process of dedifferentiation in Alzheimer’s disease that eventually results in cell death Ann N Y Acad Sci 2000 920 249 255 11193159 10.1111/j.1749-6632.2000.tb06931.x

[B18] Atamna H Heme, iron, and the mitochondrial decay of ageing Ageing Res Rev 2004 3 3 303 318 10.1016/j.arr.2004.02.002 15231238

[B19] Behl C Apoptosis and Alzheimer’s disease J Neural Transm 2000 107 11 1325 1344 10.1007/s007020070021 11145007

[B20] Björkhem I Crossing the barrier: oxysterols as cholesterol transporters and metabolic modulators in the brain J Intern Med 2006 260 6 493 508 10.1111/j.1365-2796.2006.01725.x 17116000

[B21] Boonen RA van Tijn P Zivkovic D Wnt signaling in Alzheimer’s disease: up or down, that is the question Ageing Res Rev 2009 8 2 71 82 10.1016/j.arr.2008.11.003 19101658

[B22] Bosco D Fava A Plastino M Montalcini T Pujia A Possible implications of insulin resistance and glucose metabolism in Alzheimer’s disease pathogenesis J Cell Mol Med 2011 15 9 1807 1821 10.1111/j.1582-4934.2011.01318.x 21435176 PMC3918038

[B23] Brinton RD Estrogen regulation of glucose metabolism and mitochondrial function: therapeutic implications for prevention of Alzheimer’s disease Adv Drug Deliv Rev 2008 60 13–14 1504 1511 18647624 10.1016/j.addr.2008.06.003 PMC2993571

[B24] Buhaescu I Izzedine H Mevalonate pathway: a review of clinical and therapeutical implications Clin Biochem 2007 40 9–10 575 584 17467679 10.1016/j.clinbiochem.2007.03.016

[B25] Buoso E Lanni C Schettini G Govoni S Racchi M beta-Amyloid precursor protein metabolism: focus on the functions and degradation of its intracellular domain Pharmacol Res 2010 62 4 308 317 10.1016/j.phrs.2010.05.002 20561999

[B26] Burns M Duff K Cholesterol in Alzheimer’s disease and tauopathy Ann N Y Acad Sci 2002 977 367 375 10.1111/j.1749-6632.2002.tb04839.x 12480774

[B27] Cameron B Landreth GE Inflammation, microglia, and Alzheimer’s disease Neurobiol Dis 2010 37 3 503 509 10.1016/j.nbd.2009.10.006 19833208 PMC2823849

[B28] Caricasole A Bakker A Copani A Nicoletti F Gaviraghi G Terstappen GC Two sides of the same coin: Wnt signaling in neurodegeneration and neuro-oncology Biosci Rep 2005 25 5–6 309 327 16307379 10.1007/s10540-005-2893-6

[B29] Caricasole A Copani A Caruso A Caraci F Iacovelli L Sortino MA Terstappen GC Nicoletti F The Wnt pathway, cell-cycle activation and beta-amyloid: novel therapeutic strategies in Alzheimer’s disease? Trends Pharmacol Sci 2003 24 5 233 238 10.1016/S0165-6147(03)00100-7 12767722

[B30] Chen YZ APP induces neuronal apoptosis through APP-BP1-mediated downregulation of beta-catenin Apoptosis 2004 9 4 415 422 15192323 10.1023/B:APPT.0000031447.05354.9f

[B31] Chong ZZ Li F Maiese K Stress in the brain: novel cellular mechanisms of injury linked to Alzheimer’s disease Brain Res Brain Res Rev 2005 49 1 1 21 15960984 10.1016/j.brainresrev.2004.11.005 PMC2276700

[B32] Chong ZZ Maiese K Targeting WNT, protein kinase B, and mitochondrial membrane integrity to foster cellular survival in the nervous system Histol Histopathol 2004 19 2 495 504 15024710 10.14670/hh-19.495 PMC2711548

[B33] Chorsky RL Yaghmai F Hill WD Stopa EG Alzheimer’s disease: a review concerning immune response and microischemia Med Hypotheses 2001 56 1 124 127 10.1054/mehy.2000.1148 11133269

[B34] Choy YM Lau KM Lee CY Purification and characterization of urinary choriogonadotropin from patients with hydatidiform mole J Biol Chem 1979 254 4 1159 1163 105001

[B35] Copani A Caraci F Hoozemans JJ Calafiore M Sortino MA Nicoletti F The nature of the cell cycle in neurons: focus on a “non-canonical” pathway of DNA replication causally related to death Biochim Biophys Acta 2007 1772 4 409 412 10.1016/j.bbadis.2006.10.016 17196375

[B36] Correia SC Santos RX Perry G Zhu X Moreira PI Smith MA Insulin-resistant brain state: the culprit in sporadic Alzheimer’s disease? Ageing Res Rev 2011 10 2 264 273 10.1016/j.arr.2011.01.001 21262392 PMC3056939

[B37] Coughlan CM Breen KC Factors influencing the processing and function of the amyloid beta precursor protein–a potential therapeutic target in Alzheimer’s disease? Pharmacol Ther 2000 86 2 111 145 10.1016/S0163-7258(00)00036-X 10799711

[B38] Coulson EJ Does the p75 neurotrophin receptor mediate Abeta-induced toxicity in Alzheimer’s disease? J Neurochem 2006 98 3 654 660 10.1111/j.1471-4159.2006.03905.x 16893414

[B39] Cruts M Van Broeckhoven C Loss of progranulin function in frontotemporal lobar degeneration Trends Genet 2008 24 4 186 194 10.1016/j.tig.2008.01.004 18328591

[B40] Cuello AC Bruno MA The failure in NGF maturation and its increased degradation as the probable cause for the vulnerability of cholinergic neurons in Alzheimer’s disease Neurochem Res 2007 32 6 1041 1045 10.1007/s11064-006-9270-0 17404842

[B41] Cuenda A Rousseau S p38 MAP-kinases pathway regulation, function and role in human diseases Biochim Biophys Acta 2007 1773 8 1358 1375 10.1016/j.bbamcr.2007.03.010 17481747

[B42] Das UN Acetylcholinesterase and butyrylcholinesterase as possible markers of low-grade systemic inflammation Med Sci Monit 2007 13 12 RA214 RA221 18049445

[B43] Deutsch SI Rosse RB Deutsch LH Faulty regulation of tau phosphorylation by the reelin signal transduction pathway is a potential mechanism of pathogenesis and therapeutic target in Alzheimer’s disease Eur Neuropsychopharmacol 2006 16 8 547 551 10.1016/j.euroneuro.2006.01.006 16504486

[B44] Dmitriev LF Shortage of lipid-radical cycles in membranes as a possible prime cause of energetic failure in aging and Alzheimer disease Neurochem Res 2007 32 8 1278 1291 10.1007/s11064-007-9322-0 17541743

[B45] Eckert A Keil U Marques CA Bonert A Frey C Schüssel K Müller WE Mitochondrial dysfunction, apoptotic cell death, and Alzheimer’s disease Biochem Pharmacol 2003 66 8 1627 1634 10.1016/S0006-2952(03)00534-3 14555243

[B46] Eckert A Marques CA Keil U Schüssel K Müller WE Increased apoptotic cell death in sporadic and genetic Alzheimer’s disease Ann N Y Acad Sci 2003 1010 604 609 10.1196/annals.1299.113 15033800

[B47] Farooqui AA Ong WY Farooqui T Lipid mediators in the nucleus: Their potential contribution to Alzheimer’s disease Biochim Biophys Acta 2010 1801 8 906 916 10.1016/j.bbalip.2010.02.002 20170745

[B48] Filiz G Price KA Caragounis A Du T Crouch PJ White AR The role of metals in modulating metalloprotease activity in the AD brain Eur Biophys J 2008 37 3 315 321 10.1007/s00249-007-0244-1 18270696

[B49] Florent-Béchard S Desbène C Garcia P Allouche A Youssef I Escanyé MC Koziel V Hanse M Malaplate-Armand C Stenger C The essential role of lipids in Alzheimer’s disease Biochimie 2009 91 6 804 809 10.1016/j.biochi.2009.03.004 19303044

[B50] Fraser PE Yang DS Yu G Lévesque L Nishimura M Arawaka S Serpell LC Rogaeva E St George-Hyslop P Presenilin structure, function and role in Alzheimer disease Biochim Biophys Acta 2000 1502 1 1 15 10.1016/S0925-4439(00)00028-4 10899427

[B51] Fuentealba RA Farias G Scheu J Bronfman M Marzolo MP Inestrosa NC Signal transduction during amyloid-beta-peptide neurotoxicity: role in Alzheimer disease Brain Res Brain Res Rev 2004 47 1–3 275 289 15572177 10.1016/j.brainresrev.2004.07.018

[B52] Gandy S The role of cerebral amyloid beta accumulation in common forms of Alzheimer disease J Clin Invest 2005 115 5 1121 1129 15864339 10.1172/JCI25100 PMC1087184

[B53] Gärtner U Holzer M Arendt T Elevated expression of p21ras is an early event in Alzheimer’s disease and precedes neurofibrillary degeneration Neuroscience 1999 91 1 1 5 10.1016/S0306-4522(99)00059-7 10336054

[B54] Gasparini L Xu H Potential roles of insulin and IGF-1 in Alzheimer’s disease Trends Neurosci 2003 26 8 404 406 10.1016/S0166-2236(03)00163-2 12900169

[B55] Ghosh AK Gemma S Tang J beta-Secretase as a therapeutic target for Alzheimer’s disease Neurotherapeutics 2008 5 3 399 408 10.1016/j.nurt.2008.05.007 18625451 PMC2963069

[B56] Glass CK Saijo K Winner B Marchetto MC Gage FH Mechanisms underlying inflammation in neurodegeneration Cell 2010 140 6 918 934 10.1016/j.cell.2010.02.016 20303880 PMC2873093

[B57] Goodenough S Schäfer M Behl C Estrogen-induced cell signalling in a cellular model of Alzheimer’s disease J Steroid Biochem Mol Biol 2003 84 2–3 301 305 12711016 10.1016/s0960-0760(03)00043-8

[B58] Halliday G Robinson SR Shepherd C Kril J Alzheimer’s disease and inflammation: a review of cellular and therapeutic mechanisms Clin Exp Pharmacol Physiol 2000 27 1–2 1 8 10696521 10.1046/j.1440-1681.2000.03200.x

[B59] Hamaguchi T Ono K Yamada M Anti-amyloidogenic therapies: strategies for prevention and treatment of Alzheimer’s disease Cell Mol Life Sci 2006 63 13 1538 1552 10.1007/s00018-005-5599-9 16804637 PMC11136162

[B60] Hamdane M Delobel P Sambo AV Smet C Bégard S Violleau A Landrieu I Delacourte A Lippens G Flament S Neurofibrillary degeneration of the Alzheimer-type: an alternate pathway to neuronal apoptosis? Biochem Pharmacol 2003 66 8 1619 1625 10.1016/S0006-2952(03)00533-1 14555242

[B61] Harold D Abraham R Hollingworth P Sims R Gerrish A Hamshere ML Pahwa JS Moskvina V Dowzell K Williams A Genome-wide association study identifies variants at CLU and PICALM associated with Alzheimer’s disease Nat Genet 2009 41 10 1088 1093 10.1038/ng.440 19734902 PMC2845877

[B62] Hartmann D Tournoy J Saftig P Annaert W De Strooper B Implication of APP secretases in notch signaling J Mol Neurosci 2001 17 2 171 181 10.1385/JMN:17:2:171 11816790

[B63] Hartmann T Intracellular biology of Alzheimer’s disease amyloid beta peptide Eur Arch Psychiatry Clin Neurosci 1999 249 6 291 298 10.1007/s004060050102 10653285

[B64] Hegde AN Upadhya SC The ubiquitin-proteasome pathway in health and disease of the nervous system Trends Neurosci 2007 30 11 587 595 10.1016/j.tins.2007.08.005 17950927

[B65] Hicks D John D Makova NZ Henderson Z Nalivaeva NN Turner AJ Membrane targeting, shedding and protein interactions of brain acetylcholinesterase J Neurochem 2011 116 5 742 746 10.1111/j.1471-4159.2010.07032.x 21214569

[B66] Hölscher C Li L New roles for insulin-like hormones in neuronal signalling and protection: new hopes for novel treatments of Alzheimer’s disease? Neurobiol Aging 2010 31 9 1495 1502 10.1016/j.neurobiolaging.2008.08.023 18930564

[B67] Hooper NM Roles of proteolysis and lipid rafts in the processing of the amyloid precursor protein and prion protein Biochem Soc Trans 2005 33 Pt 2 335 338 15787600 10.1042/BST0330335

[B68] Inestrosa NC Varela-Nallar L Grabowski CP Colombres M Synaptotoxicity in Alzheimer’s disease: the Wnt signaling pathway as a molecular target IUBMB Life 2007 59 4–5 316 321 17505971 10.1080/15216540701242490

[B69] Issad T Masson E Pagesy P O-GlcNAc modification, insulin signaling and diabetic complications Diabetes Metab 2010 36 6 Pt 1 423 435 21074472 10.1016/j.diabet.2010.09.001

[B70] Iwata N Saido TC Amyloid-beta peptide metabolism and Alzheimer’s disease Nihon Yakurigaku Zasshi 2003 122 1 5 14 10.1254/fpj.122.5 12843567

[B71] Johnson GV Bailey CD The p38 MAP kinase signaling pathway in Alzheimer’s disease Exp Neurol 2003 183 2 263 268 10.1016/S0014-4886(03)00268-1 14552867

[B72] Kamoun P Endogenous production of hydrogen sulfide in mammals Amino Acids 2004 26 3 243 254 15221504 10.1007/s00726-004-0072-x

[B73] Katayama T Imaizumi K Manabe T Hitomi J Kudo T Tohyama M Induction of neuronal death by ER stress in Alzheimer’s disease J Chem Neuroanat 2004 28 1–2 67 78 15363492 10.1016/j.jchemneu.2003.12.004

[B74] Kim D Tsai LH Bridging physiology and pathology in AD Cell 2009 137 6 997 1000 10.1016/j.cell.2009.05.042 19524503

[B75] Klysik J Theroux SJ Sedivy JM Moffit JS Boekelheide K Signaling crossroads: the function of Raf kinase inhibitory protein in cancer, the central nervous system and reproduction Cell Signal 2008 20 1 1 9 10.1016/j.cellsig.2007.07.003 17706925 PMC2231335

[B76] Kontush A Amyloid-beta: an antioxidant that becomes a pro-oxidant and critically contributes to Alzheimer’s disease Free Radic Biol Med 2001 31 9 1120 1131 10.1016/S0891-5849(01)00688-8 11677045

[B77] Kopan R Goate A Aph-2/Nicastrin: an essential component of gamma-secretase and regulator of Notch signaling and Presenilin localization Neuron 2002 33 3 321 324 10.1016/S0896-6273(02)00585-8 11832221

[B78] Layfield R Alban A Mayer RJ Lowe J The ubiquitin protein catabolic disorders Neuropathol Appl Neurobiol 2001 27 3 171 179 10.1046/j.1365-2990.2001.00335.x 11489136

[B79] Lee CY Landreth GE The role of microglia in amyloid clearance from the AD brain J Neural Transm 2010 117 8 949 960 10.1007/s00702-010-0433-4 20552234 PMC3653296

[B80] Lefebvre T Dehennaut V Guinez C Olivier S Drougat L Mir AM Mortuaire M Vercoutter-Edouart AS Michalski JC Dysregulation of the nutrient/stress sensor O-GlcNAcylation is involved in the etiology of cardiovascular disorders, type-2 diabetes and Alzheimer’s disease Biochim Biophys Acta 2010 1800 2 67 79 10.1016/j.bbagen.2009.08.008 19732809

[B81] Li H Wolfe MS Selkoe DJ Toward structural elucidation of the gamma-secretase complex Structure 2009 17 3 326 334 10.1016/j.str.2009.01.007 19278647 PMC2661031

[B82] Mahley RW Ji ZS Remnant lipoprotein metabolism: key pathways involving cell-surface heparan sulfate proteoglycans and apolipoprotein E J Lipid Res 1999 40 1 1 16 9869645

[B83] Mariño G López-Otín C Autophagy: molecular mechanisms, physiological functions and relevance in human pathology Cell Mol Life Sci 2004 61 12 1439 1454 15197469 10.1007/s00018-004-4012-4 PMC7079832

[B84] Masters CL Beyreuther K Alzheimer’s centennial legacy: prospects for rational therapeutic intervention targeting the Abeta amyloid pathway Brain 2006 129 Pt 11 2823 2839 17012295 10.1093/brain/awl251

[B85] Mattson MP Cellular actions of beta-amyloid precursor protein and its soluble and fibrillogenic derivatives Physiol Rev 1997 77 4 1081 1132 9354812 10.1152/physrev.1997.77.4.1081

[B86] Mattson MP Chan SL Camandola S Presenilin mutations and calcium signaling defects in the nervous and immune systems Bioessays 2001 23 8 733 744 10.1002/bies.1103 11494322

[B87] McGeer PL McGeer EG Anti-inflammatory drugs in the fight against Alzheimer’s disease Ann N Y Acad Sci 1996 777 213 220 10.1111/j.1749-6632.1996.tb34421.x 8624086

[B88] McLoughlin DM Miller CC The FE65 proteins and Alzheimer’s disease J Neurosci Res 2008 86 4 744 754 10.1002/jnr.21532 17828772

[B89] McTaggart SJ Isoprenylated proteins Cell Mol Life Sci 2006 63 3 255 267 10.1007/s00018-005-5298-6 16378247 PMC11136304

[B90] Mischoulon D Fava M Role of S-adenosyl-L-methionine in the treatment of depression: a review of the evidence Am J Clin Nutr 2002 76 5 1158S 1161S 12420702 10.1093/ajcn/76/5.1158S

[B91] Moley KH Mueckler MM Glucose transport and apoptosis Apoptosis 2000 5 2 99 105 10.1023/A:1009697908332 11232248

[B92] Morgan C Colombres M Nuñez MT Inestrosa NC Structure and function of amyloid in Alzheimer’s disease Prog Neurobiol 2004 74 6 323 349 10.1016/j.pneurobio.2004.10.004 15649580

[B93] Nagai Y Ogasawara A Heese K Possible mechanisms of A beta(1–40)- or A beta(1–42)-induced cell death and their rescue factors Nihon Yakurigaku Zasshi 2004 124 3 135 143 10.1254/fpj.124.135 15333986

[B94] Nelson TJ Alkon DL Insulin and cholesterol pathways in neuronal function, memory and neurodegeneration Biochem Soc Trans 2005 33 Pt 5 1033 1036 16246039 10.1042/BST20051033

[B95] Nixon RA A “protease activation cascade” in the pathogenesis of Alzheimer’s disease Ann N Y Acad Sci 2000 924 117 131 11193788

[B96] Pascale A Amadio M Govoni S Battaini F The aging brain, a key target for the future: the protein kinase C involvement Pharmacol Res 2007 55 6 560 569 10.1016/j.phrs.2007.04.013 17553691

[B97] Poirier J Apolipoprotein E and Alzheimer’s disease A role in amyloid catabolism. Ann N Y Acad Sci 2000 924 81 90 10.1111/j.1749-6632.2000.tb05564.x 11193807

[B98] Poirier J Apolipoprotein E, cholesterol transport and synthesis in sporadic Alzheimer’s disease Neurobiol Aging 2005 26 3 355 361 10.1016/j.neurobiolaging.2004.09.003 15639314

[B99] Puglielli L Aging of the brain, neurotrophin signaling, and Alzheimer’s disease: is IGF1-R the common culprit? Neurobiol Aging 2008 29 6 795 811 10.1016/j.neurobiolaging.2007.01.010 17313996 PMC2387053

[B100] Reid PC Urano Y Kodama T Hamakubo T Alzheimer’s disease: cholesterol, membrane rafts, isoprenoids and statins J Cell Mol Med 2007 11 3 383 392 10.1111/j.1582-4934.2007.00054.x 17635634 PMC3922347

[B101] Rogers JT Bush AI Cho HH Smith DH Thomson AM Friedlich AL Lahiri DK Leedman PJ Huang X Cahill CM Iron and the translation of the amyloid precursor protein (APP) and ferritin mRNAs: riboregulation against neural oxidative damage in Alzheimer’s disease Biochem Soc Trans 2008 36 Pt 6 1282 1287 19021541 10.1042/BST0361282 PMC2746665

[B102] Rojo LE Fernández JA Maccioni AA Jimenez JM Maccioni RB Neuroinflammation: implications for the pathogenesis and molecular diagnosis of Alzheimer’s disease Arch Med Res 2008 39 1 1 16 10.1016/j.arcmed.2007.10.001 18067990

[B103] Rosner M Hanneder M Siegel N Valli A Fuchs C Hengstschläger M The mTOR pathway and its role in human genetic diseases Mutat Res 2008 659 3 284 292 10.1016/j.mrrev.2008.06.001 18598780

[B104] Rossner S New players in old amyloid precursor protein-processing pathways Int J Dev Neurosci 2004 22 7 467 474 10.1016/j.ijdevneu.2004.07.004 15465276

[B105] Rossner S Ueberham U Schliebs R Perez-Polo JR Bigl V The regulation of amyloid precursor protein metabolism by cholinergic mechanisms and neurotrophin receptor signaling Prog Neurobiol 1998 56 5 541 569 10.1016/S0301-0082(98)00044-6 9775403

[B106] Rushworth JV Hooper NM Lipid Rafts: Linking Alzheimer’s Amyloid-β Production, Aggregation, and Toxicity at Neuronal Membranes Int J Alzheimers Dis 2010 2011 603052 21234417 10.4061/2011/603052 PMC3014710

[B107] Salminen A Ojala J Kauppinen A Kaarniranta K Suuronen T Inflammation in Alzheimer’s disease: amyloid-beta oligomers trigger innate immunity defence via pattern recognition receptors Prog Neurobiol 2009 87 3 181 194 10.1016/j.pneurobio.2009.01.001 19388207

[B108] Schöneich C Methionine oxidation by reactive oxygen species: reaction mechanisms and relevance to Alzheimer’s disease Biochim Biophys Acta 2005 1703 2 111 119 10.1016/j.bbapap.2004.09.009 15680219

[B109] Selkoe DJ Notch and presenilins in vertebrates and invertebrates: implications for neuronal development and degeneration Curr Opin Neurobiol 2000 10 1 50 57 10.1016/S0959-4388(99)00054-9 10679435

[B110] Selkoe D Kopan R Notch and Presenilin: regulated intramembrane proteolysis links development and degeneration Annu Rev Neurosci 2003 26 565 597 10.1146/annurev.neuro.26.041002.131334 12730322

[B111] Sinha S Lieberburg I Cellular mechanisms of beta-amyloid production and secretion Proc Natl Acad Sci U S A 1999 96 20 11049 11053 10.1073/pnas.96.20.11049 10500121 PMC34239

[B112] Small SA Retromer sorting: a pathogenic pathway in late-onset Alzheimer disease Arch Neurol 2008 65 3 323 328 10.1001/archneurol.2007.64 18332244 PMC2726781

[B113] Small SA Duff K Linking Abeta and tau in late-onset Alzheimer’s disease: a dual pathway hypothesis Neuron 2008 60 4 534 542 10.1016/j.neuron.2008.11.007 19038212 PMC2692134

[B114] Soreghan B Thomas SN Yang AJ Aberrant sphingomyelin/ceramide metabolic-induced neuronal endosomal/lysosomal dysfunction: potential pathological consequences in age-related neurodegeneration Adv Drug Deliv Rev 2003 55 11 1515 1524 10.1016/j.addr.2003.07.007 14597144

[B115] Stark DT Bazan NG Neuroprotectin D1 induces neuronal survival and downregulation of amyloidogenic processing in Alzheimer’s disease cellular models Mol Neurobiol 2011 43 2 131 138 10.1007/s12035-011-8174-4 21431475 PMC12866416

[B116] Tafti M Ghyselinck NB Functional implication of the vitamin A signaling pathway in the brain Arch Neurol 2007 64 12 1706 1711 10.1001/archneur.64.12.1706 18071033

[B117] Takashima A Drug development targeting the glycogen synthase kinase-3beta (GSK-3beta)-mediated signal transduction pathway: role of GSK-3beta in adult brain J Pharmacol Sci 2009 109 2 174 178 10.1254/jphs.08R29FM 19179803

[B118] Takatori Y Mechanisms of neuroprotective effects of therapeutic acetylcholinesterase inhibitors used in treatment of Alzheimer’s disease Yakugaku Zasshi 2006 126 8 607 616 10.1248/yakushi.126.607 16880719

[B119] Toledo EM Colombres M Inestrosa NC Wnt signaling in neuroprotection and stem cell differentiation Prog Neurobiol 2008 86 3 281 296 10.1016/j.pneurobio.2008.08.001 18786602

[B120] Townsend KP Obregon D Quadros A Patel N Volmar C Paris D Mullan M Proinflammatory and vasoactive effects of Abeta in the cerebrovasculature Ann N Y Acad Sci 2002 977 65 76 10.1111/j.1749-6632.2002.tb04799.x 12480734

[B121] Turner PR O’Connor K Tate WP Abraham WC Roles of amyloid precursor protein and its fragments in regulating neural activity, plasticity and memory Prog Neurobiol 2003 70 1 1 32 10.1016/S0301-0082(03)00089-3 12927332

[B122] Uthayathas S Karuppagounder SS Thrash BM Parameshwaran K Suppiramaniam V Dhanasekaran M Versatile effects of sildenafil: recent pharmacological applications Pharmacol Rep 2007 59 2 150 163 17556793

[B123] Van Nostrand WE Melchor J Wagner M Davis J Cerebrovascular smooth muscle cell surface fibrillar A beta. Alteration of the proteolytic environment in the cerebral vessel wall Ann N Y Acad Sci 2000 903 89 96 10.1111/j.1749-6632.2000.tb06354.x 10818493

[B124] Vasto S Candore G Listì F Balistreri CR Colonna-Romano G Malavolta M Lio D Nuzzo D Mocchegiani E Di Bona D Inflammation, genes and zinc in Alzheimer’s disease Brain Res Rev 2008 58 1 96 105 10.1016/j.brainresrev.2007.12.001 18190968

[B125] Vetrivel KS Thinakaran G Membrane rafts in Alzheimer’s disease beta-amyloid production Biochim Biophys Acta 2010 1801 8 860 867 10.1016/j.bbalip.2010.03.007 20303415 PMC2886169

[B126] Wang LH Besirli CG Johnson EM Mixed-lineage kinases: a target for the prevention of neurodegeneration Annu Rev Pharmacol Toxicol 2004 44 451 474 10.1146/annurev.pharmtox.44.101802.121840 14744254

[B127] Weggen S Rogers M Eriksen J NSAIDs: small molecules for prevention of Alzheimer’s disease or precursors for future drug development? Trends Pharmacol Sci 2007 28 10 536 543 10.1016/j.tips.2007.09.004 17900710

[B128] Wei J Bhattacharyya S Varga J Peroxisome proliferator-activated receptor γ: innate protection from excessive fibrogenesis and potential therapeutic target in systemic sclerosis Curr Opin Rheumatol 2010 22 6 671 676 10.1097/BOR.0b013e32833de1a7 20693905 PMC4536822

[B129] Wellington CL Hayden MR Caspases and neurodegeneration: on the cutting edge of new therapeutic approaches Clin Genet 2000 57 1 1 10 10733228 10.1034/j.1399-0004.2000.570101.x

[B130] Woodgett JR Judging a protein by more than its name: GSK-3 Sci STKE 2001 2001 100 re12 11579232 10.1126/stke.2001.100.re12

[B131] Wu HY Tomizawa K Matsui H Calpain-calcineurin signaling in the pathogenesis of calcium-dependent disorder Acta Med Okayama 2007 61 3 123 137 17593948 10.18926/AMO/32905

[B132] Wyss-Coray T Rogers J Inflammation in Alzheimer Disease−−A Brief Review of the Basic Science and Clinical Literature Cold Spring Harbor Perspect Med 2012 2 1 a006346 10.1101/cshperspect.a006346 PMC3253025 22315714

[B133] Yamamoto Y Gaynor RB Therapeutic potential of inhibition of the NF-kappaB pathway in the treatment of inflammation and cancer J Clin Invest 2001 107 2 135 142 10.1172/JCI11914 11160126 PMC199180

[B134] Yang SY He XY Miller D HSD17B10: a gene involved in cognitive function through metabolism of isoleucine and neuroactive steroids Mol Genet Metab 2007 92 1–2 36 42 17618155 10.1016/j.ymgme.2007.06.001

[B135] Yoshimura T Arimura N Kaibuchi K Molecular mechanisms of axon specification and neuronal disorders Ann N Y Acad Sci 2006 1086 116 125 10.1196/annals.1377.013 17185510

[B136] Zhao WQ Townsend M Insulin resistance and amyloidogenesis as common molecular foundation for type 2 diabetes and Alzheimer’s disease Biochim Biophys Acta 2009 1792 5 482 496 10.1016/j.bbadis.2008.10.014 19026743

[B137] Zipp F Waiczies S Aktas O Neuhaus O Hemmer B Schraven B Nitsch R Hartung HP Impact of HMG-CoA reductase inhibition on brain pathology Trends Pharmacol Sci 2007 28 7 342 349 10.1016/j.tips.2007.05.001 17573124

[B138] Zlokovic BV New therapeutic targets in the neurovascular pathway in Alzheimer’s disease Neurotherapeutics 2008 5 3 409 414 10.1016/j.nurt.2008.05.011 18625452 PMC2536515

[B139] Smoot ME Ono K Ruscheinski J Wang PL Ideker T Cytoscape 2.8: new features for data integration and network visualization Bioinformatics 2011 27 3 431 432 10.1093/bioinformatics/btq675 21149340 PMC3031041

