# Supplementary figures and images for: AlzPathway: a comprehensive map of signaling pathways of Alzheimer’s disease
Source: BMC Syst Biol. 2012 May 30;6:52. doi: 10.1186/1752-0509-6-52 (PMC3411424; doi:10.1186/1752-0509-6-52)

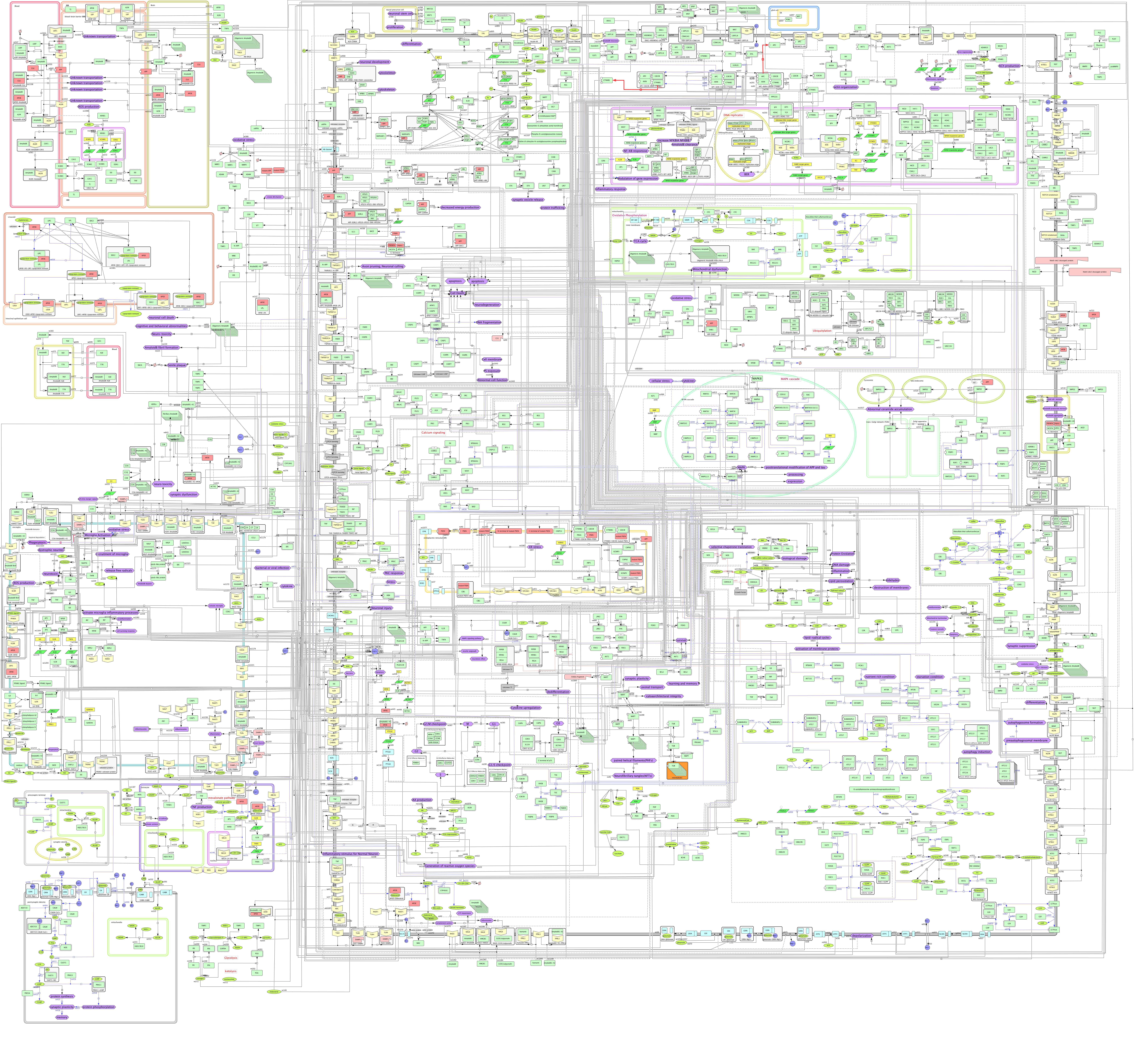

Supplement: Additional file 2: — High resolution image map of AlzPathway. The PNG file alzpathway_image_map.png contains a high resolution map of AlzPathway. This image map does not contain the reference information used for constructing the AlzPathway. SBML (CellDesigner) map and Online map (Payao) are recommended to browse the AlzPathway map. [file 1752-0509-6-52-S2.png]
