# Supplementary material for: AlzPathway: a comprehensive map of signaling pathways of Alzheimer’s disease
Source: BMC Syst Biol. 2012 May 30;6:52. doi: 10.1186/1752-0509-6-52 (PMC3411424; doi:10.1186/1752-0509-6-52)
Supplement: Additional file 3: — Instruction on usage of Payao. The PDF file alzpathway_payao_access_instruction.pdf contains insturction on usage of Payao web service (online map). Payao system requires login. AlzPathway demo user account is prepared for demo use: demo username is “ap_demo” and the corresponding password is “4patients”. As for usage of Payao system, see the user’s guide provided at Payao web site: http://payao.oist.jp:8080/payaologue/doc/PAYAO_Users_GuideE11.pdf. [file 1752-0509-6-52-S3.pdf]

Instruction on how to access the AlzPathway web service (online map) by Payao

1. Access to the AlzPathway web site (http://alzpathway.org/), and open the Pathway Map page (http://alzpathway.org/Pathway_Map.html) to see the instruction on how to access the AlzPathway web service (online map) by Payao.
2. Access to the Payao system at http://sblab.celldesigner.org:18080/Payao11/bin/.
3. Log in the Payao system by using AlzPathway demo user account:

   demo username is “**ap_demo**” and the corresponding password is “**4patients**”.

   If you do not log in, you can not access the AlzPathway online map on the Payao.

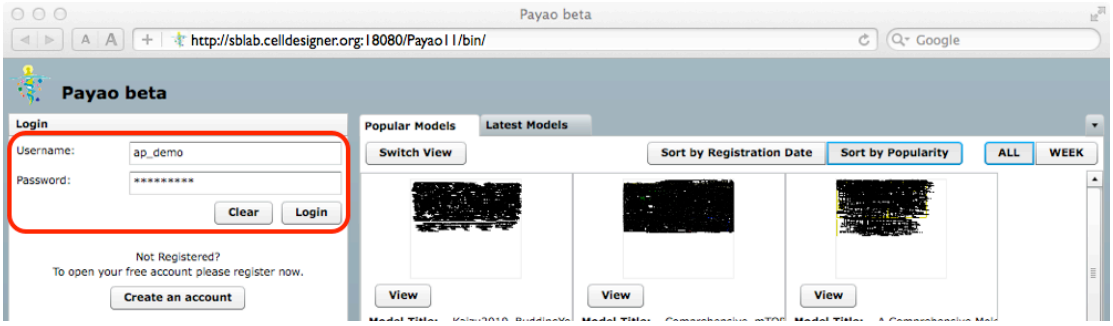

4. Click View button of AlzPathway model among Popular Models to show AlzPathway map.

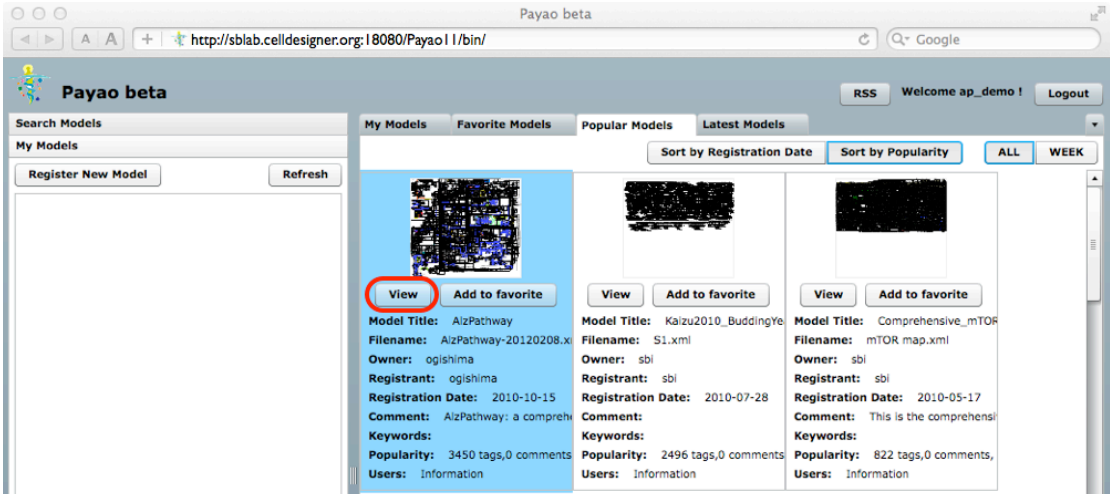

5. Browse AlzPathway map. Click a balloon to see a note (in the case of a reaction, evidences to the references in PubMed ID).

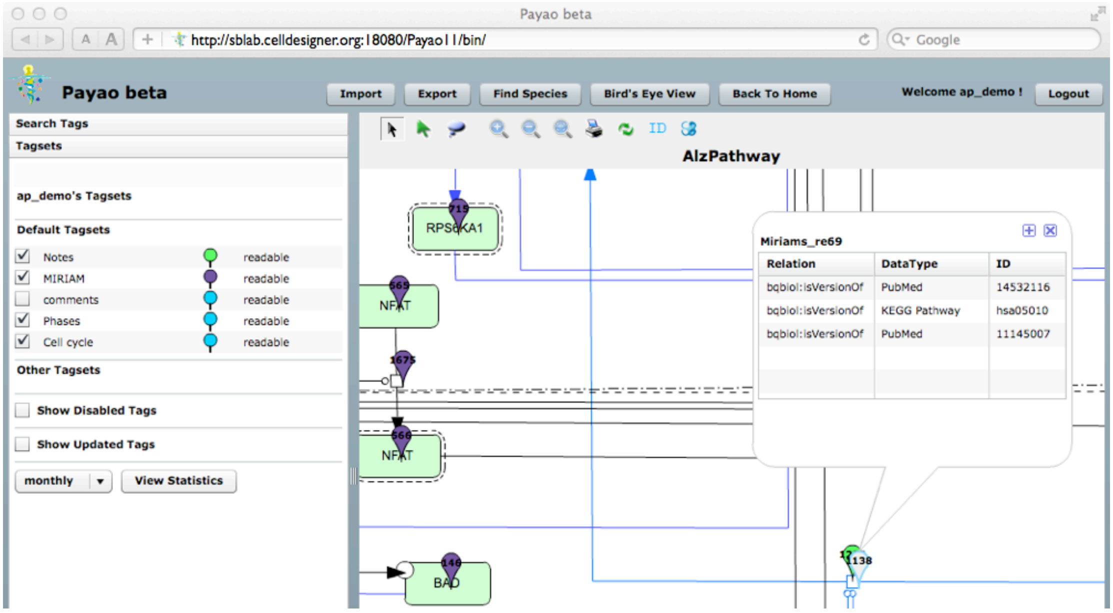

6. Browse tables of Species, Proteins, Genes, RNAs, and Reactions at the bottom of map.

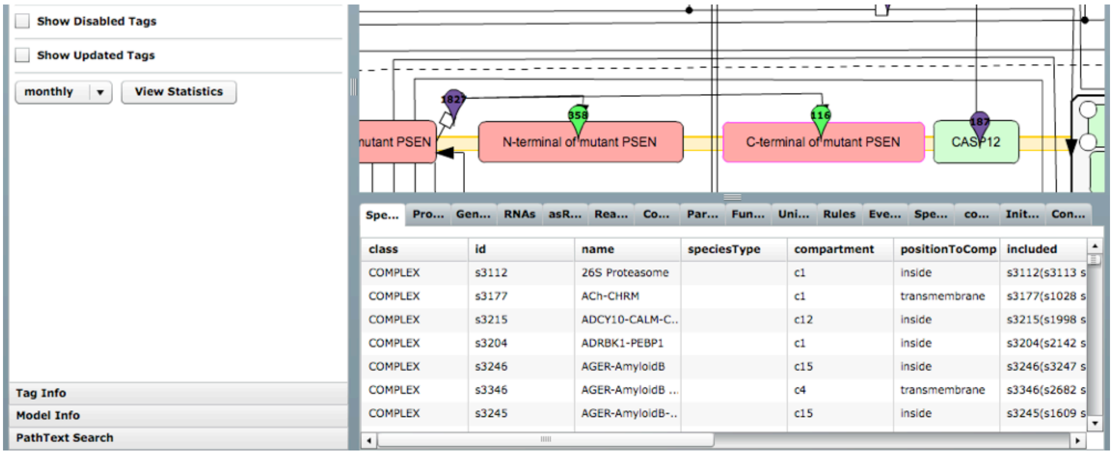

As for usage of Payao system, see the user's guide provided by Payao developers: http://payao.oist.jp:8080/payaologue/doc/PAYAO_Users_GuideE11.pdf.
